# Effect of Intermediate-Frequency Repetitive Transcranial Magnetic Stimulation on Recovery following Traumatic Brain Injury in Rats

**DOI:** 10.1155/2017/4540291

**Published:** 2017-11-29

**Authors:** Leticia Verdugo-Diaz, Francisco Estrada-Rojo, Aron Garcia-Espinoza, Eduardo Hernandez-Lopez, Alejandro Hernandez-Chavez, Carlos Guzman-Uribe, Marina Martinez-Vargas, Adan Perez-Arredondo, Tomas Calvario, David Elias-Viñas, Luz Navarro

**Affiliations:** ^1^Department of Physiology, School of Medicine, Universidad Nacional Autonoma de Mexico, Apartado Postal 70-250, 04510 Ciudad de México, Mexico; ^2^Department of Electrical Engineering, Bioelectronics Section, CINVESTAV, IPN, Av. Politecnico Nacional 2508, Col. San Pedro Zacatenco, 07360 Ciudad de México, Mexico

## Abstract

Traumatic brain injury (TBI) represents a significant public health concern and has been associated with high rates of morbidity and mortality. Although several research groups have proposed the use of repetitive transcranial magnetic stimulation (rTMS) to enhance neuroprotection and recovery in patients with TBI, few studies have obtained sufficient evidence regarding its effects in this population. Therefore, we aimed to analyze the effect of intermediate-frequency rTMS (2 Hz) on behavioral and histological recovery following TBI in rats. Male Wistar rats were divided into six groups: three groups without TBI (no manipulation, movement restriction plus sham rTMS, and movement restriction plus rTMS) and three groups subjected to TBI (TBI only, TBI plus movement restriction and sham rTMS, and TBI plus movement restriction and rTMS). The movement restriction groups were included so that rTMS could be applied without anesthesia. Our results indicate that the restriction of movement and sham rTMS per se promotes recovery, as measured using a neurobehavioral scale, although rTMS was associated with faster and superior recovery. We also observed that TBI caused alterations in the CA1 and CA3 subregions of the hippocampus, which are partly restored by movement restriction and rTMS. Our findings indicated that movement restriction prevents damage caused by TBI and that intermediate-frequency rTMS promotes behavioral and histologic recovery after TBI.

## 1. Introduction

Traumatic brain injury (TBI) is a major global health concern, representing the leading cause of brain damage in children and young adults and the most common cause of prolonged disability in Europe [1]. In the United States, more than 1.7 million individuals experience a TBI annually, and TBIs are responsible for 52,000 deaths per year [2].

TBI triggers pathological pathways that may harm brain cells via pronounced calcium entry into neurons, excitotoxicity, and formation of free radicals [3], thereby promoting neuroinflammation, neuronal death, and neurological dysfunction. Neuroprotective strategies and treatments aim to enhance neurological recovery via attenuation of these secondary lesions [4]. Indeed, it is now well known that repair (angiogenesis, axonal targeting and remodeling, remyelination, neurogenesis, and synaptogenesis) and regeneration (increased ability of pluripotent stem cells to differentiate into neurons, glia, and vascular endothelium) occur in the adult brain following TBI and that these endogenous processes can be activated exogenously [5]. At the behavioral level, the consequences of TBI may include headache, memory deficits, difficulty concentrating, and sleep disturbances [2]. Such abnormalities are due in part to the extreme sensitivity of the hippocampus to trauma [6].

Repetitive transcranial magnetic stimulation (rTMS) is a relatively novel, noninvasive method of focal cortical stimulation that is widely utilized for the investigation of cortical plasticity and cortical excitability in humans [7, 8]. Research has indicated that the localized and reversible changes in brain tissue produced by rTMS exert antidepressant properties in both humans and animal models [9]. Furthermore, the beneficial effects of rTMS on cognitive function have been demonstrated in healthy humans [10], older adults with memory dysfunction [11, 12], and patients with Alzheimer's disease [13, 14]. While the precise mechanism underlying these effects remains unknown, rTMS utilizes an electromagnet to produce a rapidly fluctuating magnetic field in the brain, which is thought to alter cerebral electrical potentials and neuronal firing patterns [7]. Several studies have further reported that rTMS induces or modulates synaptic plasticity in healthy rodents and rabbits [15, 16], reduces apoptosis in animal models of ischemia [17], and modulates intracellular calcium levels in neuron-enriched primary cortical cultures [18]. However, although several research groups have proposed the use of rTMS in patients with TBI [19–23], few studies have obtained sufficient evidence regarding the matter. For example, Pape et al. [24] utilized rTMS in a patient who had been in a coma for 287 days following TBI and in two other patients with severe TBI [25]. The authors reported no adverse effects and slight neurobehavioral improvement (based on the application of auditory evoked potentials in the first work and EEG and diverse factors like fever, change in the arterial tension, and seizure in the second work). Other researchers have utilized rTMS on only a single patient with very specific symptoms, such as auditory hallucinations [26], tinnitus [27], depression, and post-TBI alcoholism. Koski et al. [28] recently reported the use of rTMS in 15 patients who had experienced a mild TBI at least 6 months prior to their study, revealing that rTMS produced improvements in various symptoms, including headache, sleep disturbances, and cognitive deficits. Although some of these studies have been cited in a recent review [29], no reports have described the use of rTMS immediately following TBI, the time at which neuroprotective processes are most likely to occur. Furthermore, few reports have discussed the use of rTMS in animal models and these studies have all used high-frequency rTMS [30–32]. Yoon et al. [32] observed an antiapoptotic effect around the perilesional area when high-frequency rTMS (10 Hz) was implemented starting 4 days after TBI in adult rats, although no functional improvements were observed. In contrast, other researchers have observed behavioral improvements in adult [30] and immature [31] rats subjected to TBI and treated with high-frequency rTMS (10 and 20 Hz, resp.) initiated at 1 day after TBI or 9 days after TBI, respectively.

High-frequency rTMS may cause adverse effects such as stroke and seizure [33], while low-frequency rTMS has been associated with an increase in BDNF secretion [34] and reversal of A*β*1–42-mediated memory deficits in rats [35]. Therefore, studies involving the application of low- or intermediate-frequency rTMS in animal models of TBI are necessary to more fully explore the mechanism underlying the effects of TMS. In the present study, we analyzed the effect of 2 Hz frequency rTMS on behavioral and histological recovery in rats following TBI (T + TMS). We used equipment specifically designed to apply rTMS in rats, and our subjects were trained in movement restriction, allowing us to apply rTMS without the use of anesthesia. Intermediate-frequency rTMS was applied from 1 to 7 days after TBI. Our results revealed that the restriction of movement enhanced behavioral and histological recovery after TBI and that the extent of recovery was significantly greater in rats subjected to rTMS.

## 2. Materials and Methods

### 2.1. Subjects

Male Wistar rats (weight: 250–300 g) were maintained under a controlled light-dark cycle (12 : 12 h; lights on at 08:00) with ad libitum access to food and water. All animal experiments were conducted in accordance with the guidelines and approval of the local ethical committee (School of Medicine, Universidad Nacional Autonoma de Mexico). The rats were individually housed in plexiglass cages in an isolated room at a controlled temperature (23 ± 1°C).

The animals were divided into six groups: (1) control with no manipulation (C) (*n* = 8); (2) control with movement restriction and sham rTMS (R) (*n* = 9); (3) control with movement restriction and rTMS (R + TMS) (*n* = 8); (4) TBI without movement restriction (T) (*n* = 16); (5) TBI with movement restriction plus sham rTMS (T + R) (*n* = 29); (6) TBI with movement restriction plus rTMS (T + TMS) (*n* = 27). Rats in groups 4 to 6 were anesthetized prior to TBI.

### 2.2. Movement Restriction

Movement restriction was applied using a plastic cylinder (5.5 cm diameter) for 15 min a day, from 7 days prior to TBI to 7 days after TBI in groups R, R + TMS, T + R, and T + TMS.

### 2.3. TBI

Rats were anesthetized with chloral hydrate (350 mg/kg, i.p.) and subjected to TBI using an automated and previously standardized modified closed skull weight-drop injury model [37, 38]. Severe TBI was induced on the exposed and unprotected skull at the level of the motor cortex (coordinates *P* = −2 and *L* = 1.4), which had been determined using stereotaxic device as described in previous studies [39]. This model has been associated with focal damage [40], including epidural hematoma and skull fracture with or without brain damage [41]. In addition, this model reproduces acute posttraumatic hemorrhage associated with severe traumatic brain injury in humans [42]. Furthermore, MRI studies have demonstrated that this model accurately represents the clinical conditions that occur in closed skull lesions (such as those occurring in falls or motor vehicle accidents) in humans [43]. Using this model, we have obtained a mortality rate of less than 40% [39]. All experiments were performed at 13:00 during the light phase of the cycle and by the same person.

Body weight, food intake, and motor-skill behavior (using Hunter's neurobehavioral scale) were evaluated each day. We quantified mortality and bleeding immediately after TBI, while daily food intake, body weight, and neurological damage were quantified for 7 days after TBI.

### 2.4. Bleeding

External hemorrhaging was evaluated by weighing the blood drained following TBI. In brief, the blood was collected in a previously weighed paper towel, and the total weight of the blood was determined 15 min after TBI.

### 2.5. Neurological Damage

We used Hunter's 21-point behavioral-neurological scale [44] to evaluate neurological damage following TBI. We evaluated paw placement (4 points), righting reflex (1 point), horizontal-bar equilibrium (3 points), slanting platform (3 points), rotation (2 points), visual fore-paw reaching (2 points), contralateral reflex (2 points), motility (2 points), and general condition (2 points). Although this scale was designed to evaluate damage caused by cerebral ischemia, research has indicated that these two models of brain damage share many similarities in the affected pathways [45]; several previous studies have utilized this scale to investigate neurological damage in TBI [39, 46–48].

### 2.6. rTMS: The Transcranial Magnetic Stimulus Was Applied Using an In-House Electronic System (EMAGPRO 12)

This system was designed and developed by TC and DE-V at the Center of Research and Advanced Studies, IPN (Mexico City). The system consisted of a capacitor, which was charged at high voltage (maximum: 500 ± 50 V) and subsequently discharged through a figure-eight coil. A microcontroller was used to control the charge/discharge cycles, and the frequency of the stimulus could be programmed between 1 and 10 Hz. We stimulated with a frequency of 2 Hz (i.e., 2 pulses by second); thus in a minute there are 120 pulses. The duration of stimulation was 15 min per day; thus a total of 1800 pulses were administrated by day. The intensity of the pulse was 50% of the maximum output of the machine. For instance, a stimulus at a frequency of 2 Hz provided a field of 33.9 ± 10% mT when measured at a distance of 0.7 cm of the center coil. This intensity represented 120% of average resting motor threshold in all animals (as determined by visual inspection of bilateral forelimb movements).

The sham coils were of the same construction as the real coils, although they were arranged to cancel the electromagnetic stimulus. The animals received rTMS or sham stimulation beginning at 10:00 for 7 consecutive days and beginning 1 day after TBI. The movement of the animals was restricted, and a figure-eight coil designed for use in rodents (1.4 cm × 1.4 cm) was placed over the skull with direct skin contact over the site of TBI. Seven days after TBI, the animals were anesthetized with pentobarbital and transcardially perfused with 4% paraformaldehyde, following which their brains were removed and frozen as described by Caron and Stephenson, 2015 [49].

### 2.7. Histology

Rat brains were cryopreserved with 18% sucrose and sectioned into 20 *μ*m slices using a Leica cryostat. Coronal tissue sections containing exemplary dorsal hippocampus, analogous to Plate 31 of Paxinos and Watson, 1998, were collected and stained using cresyl violet.

We conducted a qualitative morphology description of the CA1 and CA3 subregions of the hippocampus and performed a cell count in photomicrographs at 10x magnification with the aid of the manual marker in Image-Pro Insight software (Media Cybernetics, Inc., Rockville, MD, USA). Cell counting was made in 3 different fields of 0.2 mm × 0.2 mm on each of 5 representative sections through the dorsal hippocampus. Counting was made by 4 different blinded subjects following these criteria: neurons with a visible nucleus and/or a complete cell contour.

We also used a scale to classify the cell dispersion of CA1, CA2, CA3, and GG. The sections were scored by 4 different blinded subjects using a semiquantitative grading system (see Table 1) as described by Shafri et al. [36].

### 2.8. Statistical Analysis

The results are reported as mean values ± standard errors of the mean (SEM). One-way analyses of variance (ANOVA) and Bonferroni post hoc analyses were performed to compare bleeding and cell counting among the groups. Two-way ANOVA and Bonferroni post hoc analyses were used to compare body weight and food intake among the groups. The Kruskal-Wallis and Kolmogorov-Smirnov *Z*-tests were used to examine differences in neurological score and cell dispersion. The level of statistical significance was set at *p* < 0.05, using Prisma Software.

## 3. Results

### 3.1. Effect of TBI and rTMS on Body Weight and Food Intake

**Figure 1:**
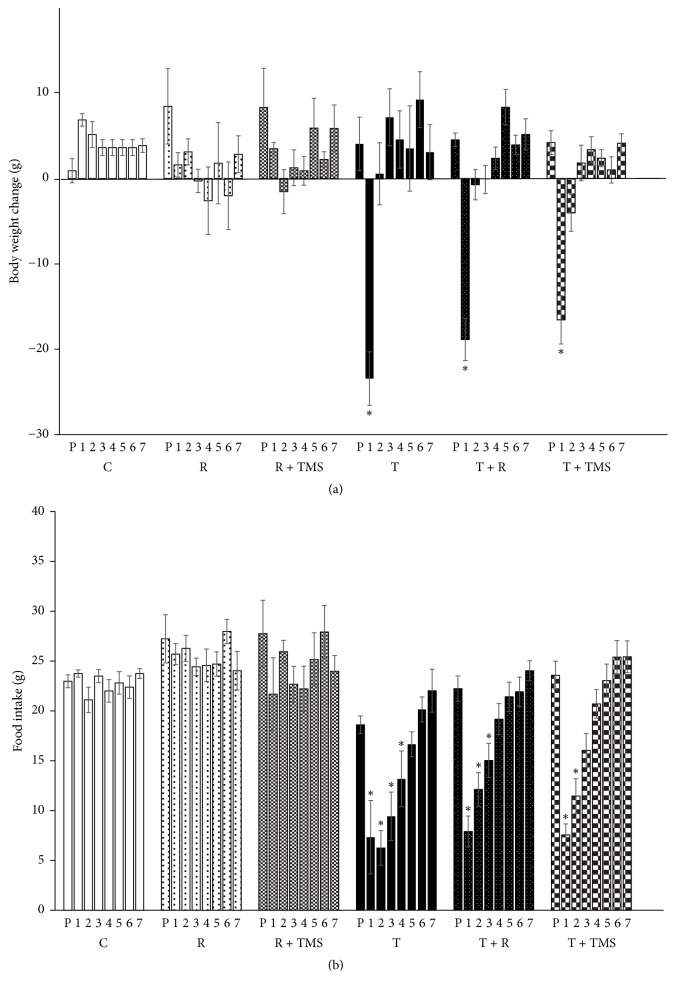
*Effect of traumatic brain injury (TBI) and repetitive transcranial magnetic stimulation (TMS) on body weight and food intake.* (a) Bars represent the mean ± SEM of daily body weight changes, measured 1 day before (P) and on days 1 to 7 after TBI. Data differed significantly according to day and experimental group; ^*∗*^*p* < 0.05 versus control group (two-way ANOVA and Bonferroni's post hoc test). (b) Bars represent the mean ± SEM of food intake (g) measured 1 day before (P) and on days 1 to 7 after TBI. Data differed significantly according to day and experimental group; ^*∗*^*p* < 0.05 versus control group (two-way ANOVA and Bonferroni's post hoc test).

TBI was associated with a significant decrease in body weight. Two-way ANOVA revealed significant differences in body weight among the experimental groups (*F*_5,590_ = 3.605; *p* < 0.003) and according to time (*F*_7,590_ = 15.738; *p* < 0.001). Body weight in group C was significantly higher from that in the T, T + R, and T + TMS groups (*p* < 0.046, 0.007, and 0.0001, resp.). Body weight 1 day after TBI was significantly lower than that on all other days. Furthermore, body weight values in groups T, T + R, and T + TMS 1 day after TBI were significantly lower than those obtained on the day prior to TBI (P) (Figure 1(a)).

A similar pattern was observed for food intake: two-way ANOVA revealed significant differences among the experimental groups (*F*_5,562_ = 37.018; *p* < 0.001) and according to time (*F*_7,562_ = 18.015; *p* < 0.001). Food intake in the C, R, and R + TMS was significantly higher from that in the T, T + R, and T + TMS groups. Moreover, food intake in the T group was significantly different from that of all other groups. Food intake on the first and second days after TBI differed significantly from that on all other days. Food intake in group T differed significantly from that in group C on days 1 through 4 after TBI, while food intake of the T + R group differed significantly from that in the R group on days 1 through 3 after TBI. Food intake in the T + TMS group differed significantly from that in the R + TMS group on days 1 and 2 after TBI (Figure 1(b)).

### 3.2. Effect of Movement Restriction on Bleeding and Mortality following Traumatic Brain Injury (TBI)

**Figure 2:**
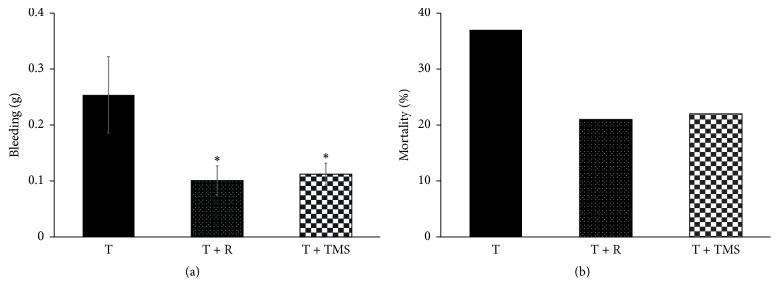
*Effect of movement restriction on bleeding and mortality following TBI.* (a) Bars represent the mean ± SEM of bleeding after TBI; ^*∗*^*p* < 0.05 (one-way ANOVA and Bonferroni's post hoc test). (b) Bars represent the mortality percentage at 8 days after TBI; *p* > 0.05, chi-square test.

We observed a significant difference in TBI-related bleeding between the T group (0.25 ± 0.07 g) and the T + R (0.10 ± 0.02 g) and T + TMS groups (0.11 ± 0.02 g) (*F*_2,69_ = 4.826; *p* < 0.001; Figure 2(a)). We also observed a difference in TBI-induced mortality, although this difference was not statistically significant (*X*^2^_2gl_ = 1.73, *p* > 0.05; Figure 2(b)).

### 3.3. Effect of TBI and TMS on Neurological Scores

**Figure 3:**
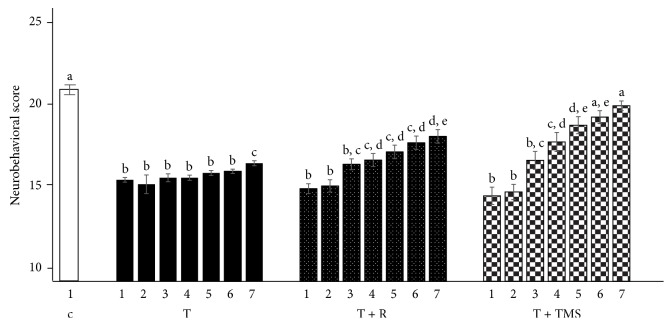
*Effect of traumatic brain injury (TBI) and repetitive transcranial magnetic stimulation (TMS) on neurological score.* Bars represent the mean ± SEM of neurological score obtained 1 day before and on days 1 to 7 after TBI. Bars labeled with the same letter represent nonsignificant differences (Kruskal-Wallis and Kolmogorov post hoc tests).

We observed significant decreases in neurobiological score from days 1 to 7 after TBI for the T and T + R groups, relative to those of the C group. In contrast, neurobiological scores in the T + TMS group differed significantly from those of the control group only on days 1 to 5 after TBI (Kruskal-Wallis *X*_ _^2^_21gl_ = 165.6, *p* < 0.001 and Kolmogorov-Smirnov *Z*-tests; Figure 3).

### 3.4. Effect of TBI and TMS on Morphological Attributes

**Figure 4:**
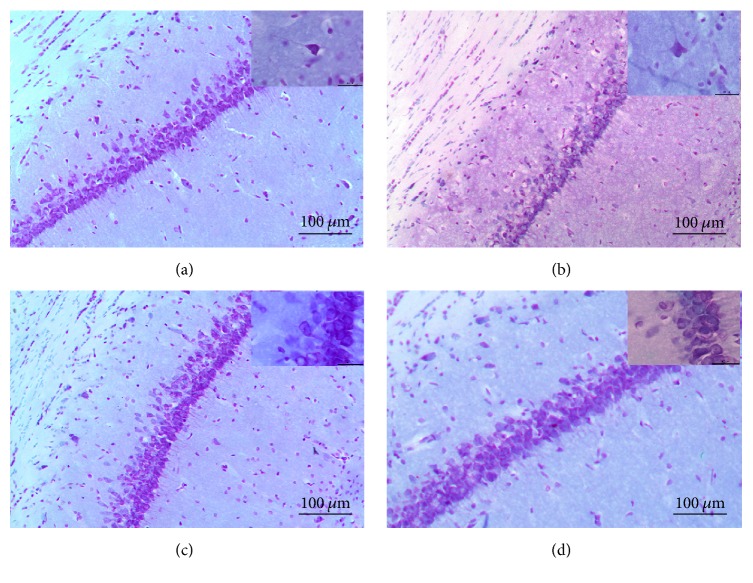
*Morphological changes in the CA1 subregion of the hippocampus represent the effect of traumatic brain injury (TBI) and repetitive transcranial magnetic stimulation (TMS)*. Photomicrographs of hippocampal area CA1 stained with cresyl violet. The magnification in the large image corresponds to 10x. Total magnification: 100x and the insets images in the upper right corner correspond to 40x. Total magnification: 100x. Sections are as follows: (a) R + TMS, (b) T, (c) T + R, and (d) T + TMS.

Morphological analysis of rat brain slices revealed qualitative modifications in the different groups. For example, the R + TMS group (Figure 4(a)) depicts the normal morphology (i.e., a polyhedral shape) of pyramidal neurons in the hippocampus: Nissl substance can be observed in the cell body, which is clear, large, and round, with a central nucleolus core. These observations suggest that the neurons are highly metabolically active. In comparison, in T group (Figure 4(b)), the organization of the pyramidal layer is lost: chromatolysis, a sign of cellular damage, can be observed, and the cell body is swollen, with a loss of Nissl substance. Furthermore, there is increased staining in the stroma around the pyramidal layer, which corresponds to leakage of the Nissl substance from the damaged cell. In T + R and T + TMS groups (Figures 4(c) and 4(d)), apparently the size of neurons is increased, probably due to cellular edema, but the organization of pyramidal neurons is conserved, with a slight loss of Nissl substance.

**Figure 5:**
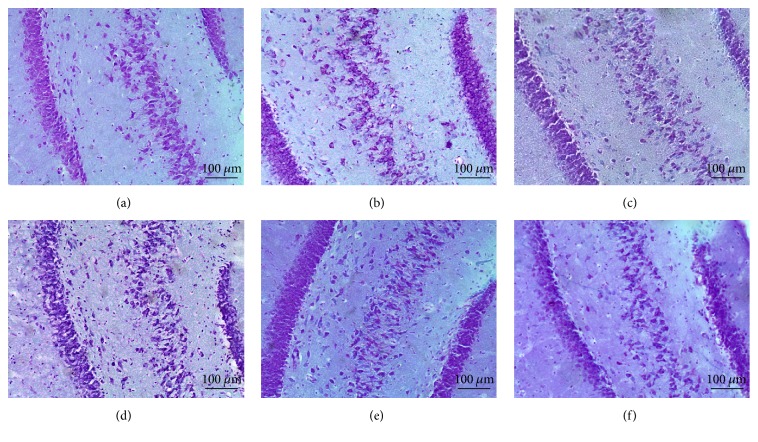
*Effect of traumatic brain injury (TBI) and repetitive transcranial magnetic stimulation (TMS) on cellular CA3 morphology*. Bright field photomicrographs of the hippocampal CA3 region stained with cresyl violet. Objective magnification: 10x. Total magnification: 100x. (a) C; (b) R; (c) R + TMS; (d) T; (e) T + R; (f) T + TMS.

We also observed an appreciable level of damage in the CA3 subregion of the hippocampus. C group (Figure 5(a)) shows that pyramidal neurons that are intact and better organized than those in T group (Figure 5(d)), which have completely lost their morphology, indicate that parts of the cells have been scattered due to nerve injury. In R group (Figure 5(b)) neuronal morphology has been preserved: clear, round, and large nuclei are visible, although a slight loss of Nissl substance relative to controls can be observed (Figure 5(a)). In R + TMS group (Figure 5(c)), there are fewer and more widely dispersed pyramidal neurons than in C group. T group and T + R (Figure 5(e)) depict greater dispersion of the pyramidal layer and fewer viable cells, with scattered cellular debris, as well as apoptotic neurons with elongated shapes and pyknotic nuclei. In T + TMS (Figure 5(f)), polyhedral viable neurons containing Nissl substance can be observed, although the Nissl substance is less apparent than in C group.

**Figure 6:**
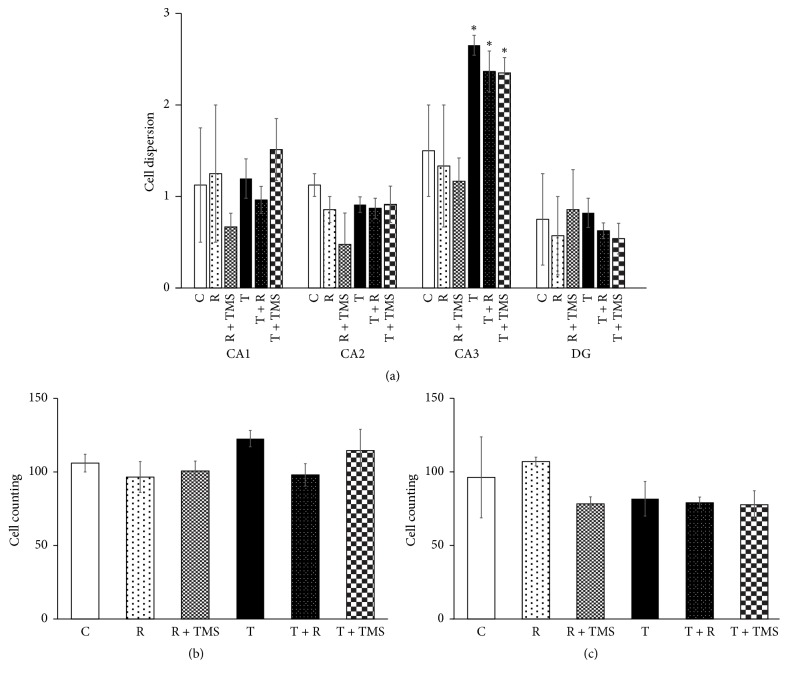
*Effect of traumatic brain injury (TBI) and repetitive transcranial magnetic stimulation (TMS) on cell dispersion and counting*. (a) Bars represent the mean ± SEM of cell dispersion in the different hippocampal subregions (CA1, CA2, CA3, and dentate gyrus [DG]). Differences were only statistically significant in the CA3 subregion; *p* < 0.05 (Kruskal-Wallis and Kolmogorov post hoc test). (b) Bars represent the sum + SEM of cell counting in 3 different fields of CA1. (c) Bars represent the sum + SEM of cell counting in 3 different fields of CA3. ^*∗*^*p* < 0.05 versus C, R, and R + TMS groups.

We observed a significant increase in cell dispersion in the CA3 subregion for groups T, T + R, and T + TMS relative to that observed in group C (Kruskal-Wallis *X*_ _^2^_12gl_ = 12.657, *p* < 0.027, and Kolmogorov-Smirnov *Z*-test, *p* < 0.05; see Figure 6(a)). Nevertheless, there were no statistically significant differences in cell counts in the CA1 and CA3 subregions of the hippocampus in any of the experimental groups, relative to group C (*F*_5,19_ = 1.344, *p* = 0.291 for CA1, and *F*_5,19_ = 0.88812, *p* = 0.5135 for CA3) (see Figures 6(b) and 6(c)).

## 4. Discussion

In the present study, we observed that TBI results in an impaired physical state in rats, which manifests as decreased food intake, resulting in decreased body weight on the first day after TBI. This decrease in food intake and body weight is part of the metabolic response to trauma [50] and has been used previously to evaluate the neuroprotective effect of various substances in brain injury models [51]. Neither movement restriction nor rTMS attenuated the decreases in body weight or food intake observed on the first day after TBI. Nevertheless, rTMS induced a less pronounced weight loss, as well as faster recovery of food intake, in rats exposed to TBI.

We further observed that TBI caused a significant deterioration in the neurobehavioral score of rats on the first several days after TBI. Partial restoration of function was observed in TBI groups at 7 days after injury. However, rats subjected to movement restriction from 7 days before TBI began to exhibit signs of recuperation on days 4–7 after TBI. We also observed that rTMS induced a significant improvement in neurobiological scores on days 4–7 after TBI, relative to those obtained 1 day after TBI. Moreover, in this experimental group, the neurobehavioral score at 6 and 7 days after TBI did not significantly differ from that of the control rats, although it did significantly differ from that of the T and T + R groups on the same days. In our previous study, we observed similar enhancements in recovery after TBI using this neurobehavioral score in conjunction with other experimental strategies, such as sleep deprivation [46].

The primary motor cortex is responsible for sensorimotor integration and precise control of voluntary movement [52]. Connections between the motor cortex and primary somatosensory cortex have been described in the literature [53, 54] and are likely to be involved in the integration of motor behavior. Thus, changes in motor behavior are thought to derive from changes in sensory experience [55], suggesting that the circuits integrated by the primary motor cortex are affected, and motor behavior is damaged, following TBI.

In the present study, neurobehavioral scores deteriorated following TBI. Nevertheless, we observed recuperation in the T + TMS group, in accordance with the findings of previous reports, which have indicated that functional and morphological changes occur after the application of extremely low-frequency electromagnetic fields [56], high-frequency rTMS [30, 31], or repetitive transcranial direct current stimulation in a rat model of stroke [57]. Furthermore, the effect of magnetic fields in the reorganization of altered neural pathways is well-documented [58, 59]. These studies have revealed that rTMS plays a role in the reorganization of abnormal neural circuits and enables functional recovery in a mouse model of visuotopic anomalies. Taken together, these findings suggest that low- or intermediate-frequency rTMS may aid in restoring neural pathways of the cortex, thereby improving motor behavior and neurobehavioral scores.

Previous studies have indicated that low frequency rTMS increases hippocampal levels of neurotrophins and NMDA receptors in a rat model of Alzheimer's disease [35]. Moreover, additional studies have revealed that low-frequency rTMS promotes the expression of c-Fos and BDNF in the cerebral cortex of rats with cerebral infarction [34]. Thus, in our TBI model, rTMS may have altered levels of various neuroprotective molecules, such as neurotrophins.

In addition, our results may be explained by an increase in cerebral blood flow in the motor and premotor areas due to 2 Hz rTMS, as demonstrated by Moisa et al. [60]. As previously mentioned, subjects must be trained to remain immobile in order to apply rTMS in awake animals. Thus, we included a group of rats in which movement restriction was maintained 15 min/day from 7 days before TBI and/or rTMS. Our results indicated that movement restriction itself promotes behavioral recovery. Moreover, bleeding was significantly lower in the movement-restricted groups than in the other groups after TBI; this finding was also correlated with decreased mortality. Although only a few studies have investigated this topic, it is possible that movement restriction promotes coagulation by increasing oxidative stress. An increase in the formation of free radicals causes an imbalance in the process of coagulation that specifically increases the number of platelets [61]; hypoxemia also causes an increase in free radicals, which triggers the activation of coagulation [62]. Acute posttraumatic sequelae include the development of coagulopathy, which is associated with increased morbidity and mortality. Such coagulopathy is accompanied by an increase in activated protein C, changes in vascular endothelial activity, hyperfibrinolysis, and platelet dysfunction [63, 64]. Research involving animal models has revealed that the decrease in platelet aggregation begins within the first 15 minutes following trauma [64] and is related to the severity of the TBI [65]. In humans, this decrease in aggregation may be present, even when platelet count remains within the normal range [66]. These findings suggest that the TBI subjects of the present study experienced posttraumatic coagulopathy that resulted in thrombocytopenia, which in turn resulted in increased bleeding. However, subjects exposed to movement restriction may have had a greater basal platelet count, which in turn allowed for faster coagulation and decreased bleeding.

Our histological analyses revealed no significant differences in cell count in the CA1, CA2, and CA3 subregions of the hippocampus. Nevertheless, movement restriction seemed to prevent gliosis and subsequent apoptosis in CA1, while rTMS prevented this in CA1 and CA3. We also observed that TBI induced disorganization in the CA3 subregion. The rodent hippocampus plays a critical role in spatial memory and navigation. In particular, the CA3 subregion plays an important role in the encoding of new spatial information within short-term memory, which persists for seconds or minutes. The CA3 subregion also functions in cooperation with the dentate gyrus in processing the geometry of the environment [67]. Deficits in learning and memory are frequently reported as consequences of TBI [68]. These findings suggest that such deficits were associated with hippocampal damage in our mouse model of TBI.

Several authors have reported disorganization in the CA3 subregion of the hippocampus in various models of brain damage (e.g., in a valproic acid rat model of autism [69], in Lis1-mutant mice [70], or after kainic acid injection in rat pups [71], which are associated with different grades of memory deficits). Although we did not explore memory in the present study, it is possible that such deficits were present in our experimental subjects.

Unlike previous rate studies involving rTMS, we utilized control animals that had been subjected to movement restriction. Thus, no anesthesia was required to apply rTMS. Moreover, we utilized intermediate-frequency rTMS, which is associated with a lower risk of side effects.

In conclusion, our findings indicated that movement restriction prevents damage caused by TBI and that intermediate-frequency rTMS promotes behavioral and histologic recovery after TBI.

## Figures and Tables

**Table 1:** Cell dispersion score in cresyl stained sections, taken from Shafri et al. [36].

Dispersion score	Description
0	Normal appearance
1	Dispersed population of cells in 1–5% area
2	Dispersed population of cells in 5–15% area
3	Dispersed population of cells in more than 15% area
